# Studying the effect of fluctuating environment on intra-atomic frequency comb based quantum memory

**DOI:** 10.1038/s41598-021-90945-6

**Published:** 2021-06-01

**Authors:** G. P. Teja, Sandeep K. Goyal

**Affiliations:** Indian Institute of Science Education and research, Mohali, Punjab 140306 India

**Keywords:** Quantum physics, Quantum mechanics, Physics

## Abstract

In this article, we study the effect of various environmental factors on intra-atomic frequency comb (I-AFC) based quantum memory. The effect of the environment is incorporated as random fluctuations and non-uniformity in the parameters such as comb spacing and the optical depth, of the frequency comb. We found that the I-AFC is viable for photon storage even for very large fluctuations in the parameters of the frequency comb, which makes I-AFC a robust platform for photon storage. Furthermore, we show that the non-uniform frequency combs without any fluctuations in the comb parameters can also yield efficient quantum memory. Since the intra-atomic frequency combs found in natural atomic systems are often non-uniform, our results suggest that a large class of these systems can be used for I-AFC based efficient quantum memory.

## Introduction

Quantum memory is a device which can store and reemit photons on demand. Quantum memory is essential for photonic quantum information processing and long-distance quantum communications^1–3^. Along with probabilistic single-photon sources, it can also be used to achieve deterministic single-photon sources^4,5^. Among the atomic ensemble based quantum memories electromagnetically induced transparency^6–12^, controlled reversible inhomogeneous broadening^13–18^, gradient echo memory^19–22^, Raman quantum memory^23–26^ and the atomic frequency combs (AFCs)^27–33^ are the most prevalent protocols for photonic quantum memory. The basic idea behind an atomic ensemble based quantum memory is the controlled reversible transfer of information between the light field and the atomic states. The incoming photons are made to interact with the ensemble of atoms and the excitation is transferred to a long-lived state. Reversing the process results in the photon emission.

Atomic frequency comb based quantum memory relies in artificially created frequency comb by reshaping the inhomogeneously broadened spectrum by means of optical hole burning in an ensemble of atoms. The incoming photon is absorbed as a delocalized excitation over the frequency comb. The comb-like structure of the atomic spectrum results in a photon-echo at a later time; hence the frequency comb serves as a delay line for the photon. To achieve an on-demand quantum memory, the excitation can be transferred to a long-lived spin state by applying an appropriate $\pi$-pulse. By applying another $\pi$-pulse the excitation can be transferred back to the excited state which will be emitted in the photon-echo.

In the AFC based quantum memory, the photons are stored as a delocalized excitation over all the teeth of the frequency comb which consist of billions of atoms. Therefore, in order for AFC to work the entire atomic ensemble must behave like a single quantum system. A small relative fluctuation in the frequencies of different atoms may give rise to strong decoherence in the frequency comb resulting in no photon-echo. This restricts the temperature range of AFC based quantum memory to a few Kelvins.

The intra-atomic frequency comb (I-AFC) based quantum memory is operationally similar to the one using AFC^33^. The difference being that the frequency comb in I-AFC is constructed using the degenerate hyperfine energy levels of an atom. The degeneracy in the hyperfine levels is lifted by applying external magnetic field. However, there are certain limitations of I-AFC which may affect the quality of quantum memory.

One of the limitation of the I-AFC is that the frequency combs obtained in natural atomic systems are not always uniform. The non-uniformity can be due to unequal absorption or due to unequal spacing between different hyperfine states. This may severely affect the storage quality, forcing us to choose the atomic systems which have almost uniform frequency combs. Further, the local fluctuations in the electric and magnetic fields can cause fluctuations in various parameters of the frequency comb. For example, the stray electric field can shift the frequency comb. Therefore, random fluctuations in the electric field can give rise to broadening of the transition lines. Similarly, fluctuating magnetic field can affect the spacing between different transitions. We can characterize the non-uniformities as internal, which do not have any fluctuations, and external which is due to the fluctuating environment. Both of these internal and external non-uniformities can cause low-quality for photon storage. In this article, we numerically study the effect of all these adversities on the quality of the I-AFC based quantum memory. Since, fluctuating environment affects the absorption, the line width, and the mean frequency of the transition, we study the effect of fluctuation in these quantities without considering any specific model for the environment. The effect of random fluctuations in the absorption and the comb spacing is incorporated by introducing randomness in the said parameters stochastically and then the macroscopic polarization is obtained by averaging over the fluctuations.

We characterize the quality of the quantum memory on the bases of its storage efficiency and the fidelity between the input and output state. Our study shows that the fluctuations in different parameters in the I-AFC affect the quality of the photon storage differently. For example, the fluctuations in the absorption in different teeth of the comb, i.e., non-uniformity in the height of the teeth has negligible effect on the efficiency. Whereas the fluctuations in the comb spacing has significant effect. Fortunately, this adverse effect can be mitigated by increasing the finesse of the frequency comb, which can be done by increasing the external magnetic field. Similarly, the fidelity between the input and the output states is robust against the fluctuation in the absorption and the comb spacing. Therefore, our study conclusively establish that a large class of atomic systems can be used as photonic quantum memory using I-AFC protocol.

The article is organized as follows: in Section “Background” we introduce the concepts useful to understand our results and calculations. Here we briefly explain the concept of AFC and I-AFC based quantum memory and the calculations for photon-echo efficiencies in the forward and backward propagation. In Section “Results” we present our results and numerical simulations for the effect of random environment on the efficiency in I-AFC. We conclude in Section “Conclusion”.

## Background

In this section, we discuss the topics which are relevant for results presented in this article. We start by discussing the AFC protocol for quantum memory followed by I-AFC protocol.

### AFC

**Figure 1:**
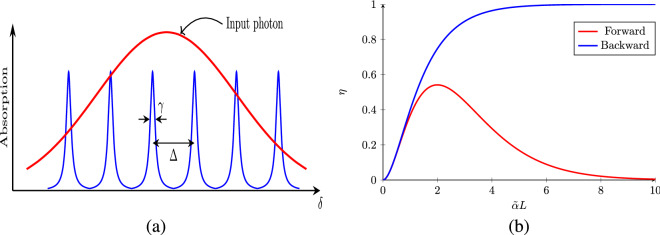
(**a**) AFC with tooth width $\gamma$ and comb spacing $\Delta$. The red curve shows the spectrum of width $\gamma _p$ of the incoming photon which interacts with the AFC. For the AFC to work efficiently $\gamma _p \gg \Delta$. (**b**) Forward and backward efficiency $\eta$ for AFC as a function of optical depth $\tilde{\alpha }L$.

AFC typically consists of rare-earth ions doped in a dielectric crystal^27,28,30,31,34–36^. The rare-earth ions when doped in crystals experience in-homogeneously broadened spectral lines due to their interaction with the local environment in the host material. This spectrum can be reshaped by applying narrow-band lasers to transfer a fraction of population of ions corresponding to a chosen frequency to a stable auxiliary state, resulting in a hole in the spectrum. Repeating the process at desired frequencies reshapes the spectrum to yield a comb like structure (Fig. 1a). In a uniform AFC, the spacing between the neighboring teeth of the comb is fixed (say $\Delta$) and every tooth has a width of $\gamma$. In the approximation that $\gamma \ll \Delta$ the frequency of the *n*-th tooth can be written as $\delta _n = \omega _0 + n\Delta$ around some mean frequency $\omega _0$. The total size of the frequency comb is $\Gamma = N\Delta \gg \Delta$ for *N* number of teeth.

When a photon of spectral width $\gamma _p \gg \Delta$ is absorbed in the AFC with *N* number of teeth, the state of the AFC can be formally written as^37,38^

1 $$\begin{aligned} |\Psi \rangle _{\text {AFC}}&= \sum _{j=1}^N \left( c_je^{\mathrm {i}\delta _j t}|\{e_j\}\rangle \prod _{k\ne j} |\{g_k\}\rangle \right) . \end{aligned}$$

Here $|\{g_j\}\rangle$ and $|\{e_j\}\rangle$ represent the ground and collective single-excitation state of all the atoms with detuning $\delta _j$, respectively, and the $c_j$’s represent the absorption coefficient of each tooth in the comb. The probability of photon emission from the AFC is $P(t) = |\sum _{j=1}^{N} c_j e^{\mathrm {i}j t \Delta }|^2$ where we have used $\delta _j = j\Delta$. The *P*(*t*) vanishes at all times *t* except for $t_n = 2n\pi /\Delta$ for positive integers *n* resulting in *n*-th photon-echo.

The collective dynamics of electric field and the atomic state of the AFC in the weak field approximation is governed by the following Maxwell–Bloch equations^8,39^

2 $$\begin{aligned} \frac{\partial \rho _{eg}}{\partial t}&= (-\mathrm {i}\delta -\dfrac{\gamma }{2}) \rho _{eg} + \mathrm {i}\dfrac{d_{eg}}{2\hbar } {\mathcal {E}}(z,t), \end{aligned}$$

3 $$\begin{aligned} \left( \frac{\partial }{\partial t} \pm c\frac{\partial }{\partial z} \right) {\mathcal {E}}(z,t)&= \mathrm {i}\dfrac{\omega _L d_{ge}}{ \epsilon _0 V} \int n(\delta ) \rho _{eg}(z,t,\delta ) \,\text {d}\delta . \end{aligned}$$

Here $n(\delta )$ is the atomic spectral distribution which characterize the AFC, $\delta =\omega _{eg}-\omega _L$ is the detuning between light $\omega _L$ and atomic transition $\omega _{eg}$. The $\rho _{eg} \equiv \rho _{eg}(z,t)$ is off-diagonal density matrix element, $d_{eg}$ is the transition dipole matrix element for the transition $|e\rangle$-$|g\rangle$. The ± sign in Eq. (3) represent the forward and backward propagating modes of light. For the case of forward propagating modes, solving these equations yields the output electric field ${\mathcal {E}}_f$ as a function of *z* and the frequency $\omega$, which reads

4 $$\begin{aligned} {\mathcal {E}}_f(z,\omega )= {\mathcal {E}}_f(0,\omega )e^{-\mathcal {D}z}. \end{aligned}$$

The input and the output electric field are related by the propagator $\mathcal {D}$ which is given by

5 $$\begin{aligned} \mathcal {D} =\alpha \int \dfrac{n(\delta )}{\mathrm {i}(\omega + \delta )+\gamma /2} \,\text {d}\delta +\dfrac{\mathrm {i}\omega }{c},&\alpha = \dfrac{{|d_{eg}|}^{2}\omega _{L}}{2\hbar \epsilon _0 c V}. \end{aligned}$$

Here $\alpha$ is the absorption coefficient and *V* is the volume of the atomic ensemble.

In Principle, the electric field in the time domain ${\mathcal {E}}_f(z,t)$ can be calculated by taking the inverse Fourier transform of ${\mathcal {E}}_f(z,\omega )$; however, the expression for the same will be very cumbersome. In the limit $\Gamma \gg \gamma _p\gg \Delta$, and after propagating the electric field for a distance *L*, a simplified expression for ${\mathcal {E}}_f(L,t)$ can be written as ^28^

6 $$\begin{aligned} {\mathcal {E}}_f(L,t)&= e^{- \left( \sqrt{2}\pi /{\mathcal {F}}\right) ^2} \left( \tilde{\alpha } L e^{-\tilde{\alpha } L/2}\right) \, {\mathcal {E}}_f(0,t-2\pi /\Delta ), \end{aligned}$$

with $\tilde{\alpha }=\alpha /{\mathcal {F}}$ and the finesse ${\mathcal {F}}$ of AFC is the ratio between comb spacing and tooth width, i.e., ${\mathcal {F}}=\Delta /\gamma$.

The AFC on its own results in a photon-echo which can be thought of as a delay line which is typically of the order of microseconds and solely depends on the comb spacing $\Delta$. In order to achieve on-demand retrieval of the input photon the excitation is transferred from the $|e\rangle$ state to a long-lived spin state $|s\rangle$ by applying a $\pi$-pulse. After the storage time (limited by the lifetime of the spin state $|s\rangle$) a second $\pi$-pulse is applied to transfer the excitation back to $|e\rangle$ which due to AFC will re-phase after time $2\pi /\Delta$^30^. Thus, one can achieve an on-demand and deterministic quantum memory.

Application of two $\pi$-pulses results in the overall sign change in the electric field which causes backward propagation of light. If we apply the $\pi$-pulses at time $t=\pi /\Delta$ the atomic polarization induced by the input electric field will act as the source term for the backward field propagation. On solving the Maxwell–Bloch equations, the electric field in the backward mode can be written as^28^

7 $$\begin{aligned} {\mathcal {E}}_b(L,t)&= e^{- \left( \sqrt{2}\pi /{\mathcal {F}}\right) ^2} (1-e^{-\tilde{\alpha } L})\,{\mathcal {E}}_f(0,t-2\pi /\Delta ), \end{aligned}$$

which is same as forward mode solution apart from the factor $1-e^{-\tilde{\alpha } L}$ instead of $\tilde{\alpha } L e^{-\tilde{\alpha } L/2}$.

The quality of the quantum memory is characterized by the efficiency $\eta$ and the fidelity $\mathfrak {F}$. The efficiency is defined as the ratio of the intensity of light in the first echo to the input light, which determines the probability of the retrieving the photon from the atomic ensemble. On the other hand the fidelity is defined as the overlap between the input pulse and the first echo, which determines the change in the state of the stored photon. The expressions for $\eta$ and the $\mathfrak {F}$ are given by

8 $$\begin{aligned} \eta = \dfrac{\int _{\pi /\Delta }^{3\pi /\Delta } {|{\mathcal {E}}(L,t)|}^2 \, dt }{ \int {|{\mathcal {E}}(0,t)|}^2\, dt}, \quad \mathfrak {F}= \dfrac{{|\int _{\pi /\Delta }^{3\pi /\Delta } {\mathcal {E}}^*(0,t-{2\pi }/{\Delta }) ~{\mathcal {E}}(L,t) \, dt|}^2 }{ \int {|{\mathcal {E}}(0,t-{2\pi }/{\Delta })|}^2\, dt ~ \int _{\pi /\Delta }^{3\pi /\Delta } {|{\mathcal {E}}(L,t)|}^2 \, dt }. \end{aligned}$$

For high finesse (${\mathcal {F}} \gg 1$) the efficiency for the forward propagating modes of light becomes $\eta _f = (\tilde{\alpha } L)^2 \exp (-\tilde{\alpha } L)$ which can approach to a maximum value of $54\%$ for $\tilde{\alpha }L\equiv \alpha L/{\mathcal {F}}=2$ (Fig. 1b). Since ${\mathcal {F}}\gg 1$ the forward mode requires high absorption ($\alpha L > {\mathcal {F}}$) for maximum efficiency. The efficiency in the backward-mode is $\eta _b(L) = (1-e^{-\tilde{\alpha } L})^2$ which can be optimized over absorption and finesse to reach $100\%$ (Fig. 1b).

In order for AFC to work as a quantum memory, a large number the atoms in the atomic ensemble need to work as a single macroscopic quantum system. The incoming photon is absorbed as a collective excitation over all the atoms coherently. Any small phase fluctuation between these atoms will cause the AFC to fail. This makes the AFC protocol for quantum memory very sensitive to local environment. To overcome this problem one can exploit the degeneracy in the atomic states of alkali atoms to realize a frequency comb. Such frequency combs are called I-AFC, which is introduced in the following subsection.

### I-AFC

In I-AFC we start by considering an optical transition between hyper-fine degenerate energy levels $\{|g_m\rangle \}$ and $\{|e_n\rangle \}$ of an alkali atom. The degeneracy in the excited and ground states is lifted by applying external magnetic field with splitting proportional to magnetic field. Collectively, all the dipole allowed transitions between the ground state manifold and the excited state manifold yield a comb like structure similar to the one in Fig. 1a, which is known as I-AFC^33^.

The propagation of electromagnetic field through an ensemble of atom possessing I-AFC can be calculated using the Maxwell–Bloch equations. The propagator ${\mathcal {D}}$ and absorption coefficient $\alpha$ for the dynamics reads

9 $$\begin{aligned} \mathcal {D} = \sum _{nm} \dfrac{\alpha _{nm}}{1/2 + \mathrm {i}/\gamma (n \Delta +\omega )}+\dfrac{\mathrm {i}\omega }{c}, \qquad \alpha _{nm} =\mathcal {N} \rho _{mm} \dfrac{{|d_{nm}|}^{2}\omega _{L}}{2\hbar \epsilon _0 c \gamma }. \end{aligned}$$

Here ${\mathcal {E}}(0,\omega )$ is the input pulse with the mean frequency $\omega _L$, and $\gamma$, $\mathcal {N}$ and $d_{nm}$ are the tooth width, number density of atoms and the transition dipole moment between *n*th excited and *m*th ground state, respectively.

Similar to AFC, the dynamics of the electric field in I-AFC is completely characterized by the propagator ${\mathcal {D}}$, which inturn is controlled by the finesse ${\mathcal {F}}$ and absorption coefficient $\alpha$. Despite the differences between the propagators of AFC and I-AFC [Eqs. (5) and (9)], they yield similar results for photon-echo and efficiencies favoring high finesse and optical depths^33^.

The calculations for the forward efficiency $\eta _f$ in I-AFC can be done by calculating the ratio between the intensities in first echo and the total input intensity, as given in Eq. (8). However, the efficiency for the back scattering $\eta _b$ requires an indirect approach^33^. In order to calculate $\eta _b$, first we estimate the average $\tilde{\alpha }$ for I-AFC by comparing the I-AFC data with the AFC data for the forward propagation. By comparing forward efficiencies of I-AFC with the Eq. (6) we obtain the common overall factor ($e^{- \left( \sqrt{2}\pi /{\mathcal {F}}\right) ^2}$) which along with $\tilde{\alpha }$ is used to calculate the backward efficiencies.

If the atomic ensemble is at temperature *T*, then the effect of temperature can be incorporated by adding Doppler shifts ${\mathbf{k}\cdot \mathbf{v}}$ to detunings $\delta$ i.e. $\delta \rightarrow \delta -{\mathbf{k}.\mathbf{v}}$ where $\mathbf{v}$ is the thermal velocity of the atoms and $\mathbf{k}$ is the wavevector of the incoming light. Then the macroscopic polarization is obtained by taking average over the entire velocity range with the corresponding probability distribution $P(\mathbf{v}) \propto \exp (-mv^2/2k_bT)$, i.e.,

10 $$\begin{aligned} \mathcal {P}( \omega )&= {\mathcal E}( \omega ) \int \mathcal {D}( \omega ,\mathbf{v}) ~P(\mathbf{v}) ~ d\mathbf{v}. \end{aligned}$$

Here $k_b$ is the Boltzman constant and *m* is the mass of the atom. For simplicity we approximate the Gaussian probability distribution with a Lorentzian distribution^33^ to obtain an analytic expression for the propagator $\mathcal {D}( \omega )$ as

11 $$\begin{aligned} \mathcal {D}( \omega )&= \sum _{n} \dfrac{\gamma \alpha _n}{\gamma /2+\Gamma _T+\mathrm {i}(\delta _{n}+\omega )}+\dfrac{\mathrm {i}\omega }{c}, \end{aligned}$$

where $\Gamma _T$ is the Doppler broadening at temperature *T* and the finesse ${\mathcal {F}}$ is redefined with Doppler width as ${\mathcal {F}}=\dfrac{\Delta }{\gamma +2 \Gamma _T}$. For the rest of the article we set $T=10$K and finally Eq. (11) results in the output electric^33^

12 $$\begin{aligned} {\mathcal {E}}(L,\omega )= {\mathcal {E}}(0,\omega )e^{-\mathcal {D}L}. \end{aligned}$$

For simplicity we have assumed here that the spacing between the teeth of the comb $\Delta$ is uniform. In real system such as Cesium atoms, the comb spacing (controlled by external magnetic field) and the absorption coefficient of each of the tooth can be non-uniform. Furthermore, local fluctuations in the electric or magnetic fields can give rise to fluctuations in the teeth spacing and variation in the height of each teeth of the frequency comb. These factors can result in non-optimum quality of the quantum memory. In the subsequent section, we study the effect of non-uniformity and local fluctuations on the efficiency of I-AFC based quantum memory. As an example, we also consider the I-AFC in the Cesium atom.

## Results

Although, the propagation of light through I-AFC and AFC is identical, in physical scenario the efficiencies can be very different for the two. This is due to the fact that in AFC a uniform frequency comb can be constructed on demand and as desired; however, in I-AFC the shape of the comb is determined by the atomic structure. In general, naturally available frequency combs are not uniform. Moreover, the fluctuations in the applied magnetic field and spatial distribution of the atoms in the ensemble may also result in non-uniform frequency comb. There are several other factors which can cause low efficiency in I-AFC. In this section, we address the effects of (i) random and non-uniform comb spacing, (ii) random and non-uniform optical depth on the efficiency of quantum memory.

The random effects are incorporated in the macroscopic polarization by averaging over the fluctuating polarization with a random parameter $\zeta$ which occurs with probability distribution $P(\zeta )$. The formal expression for the average polarization reads

13 $$\begin{aligned} \mathcal {P}( \omega ) = \tilde{\mathcal E}( \omega ) \int \mathcal {D}( \omega ,\zeta ) ~P(\zeta ) ~ d\zeta . \end{aligned}$$

For the purpose of calculations, we consider an I-AFC with a total of seven teeth at temperature $T = 10$ K and light pulse with Gaussian spectrum of the following form:

14 $$\begin{aligned} \mathcal {\tilde{E}}(0,\omega )= \exp \left[-\dfrac{\omega ^2}{2(2\Delta )^2} \right], \end{aligned}$$

where $\Delta$ is the average comb spacing. The total spectral width of the photon is chosen in such a way that it covers all the seven peaks of the frequency comb.

### Random and non-uniform comb spacing

**Figure 2:**
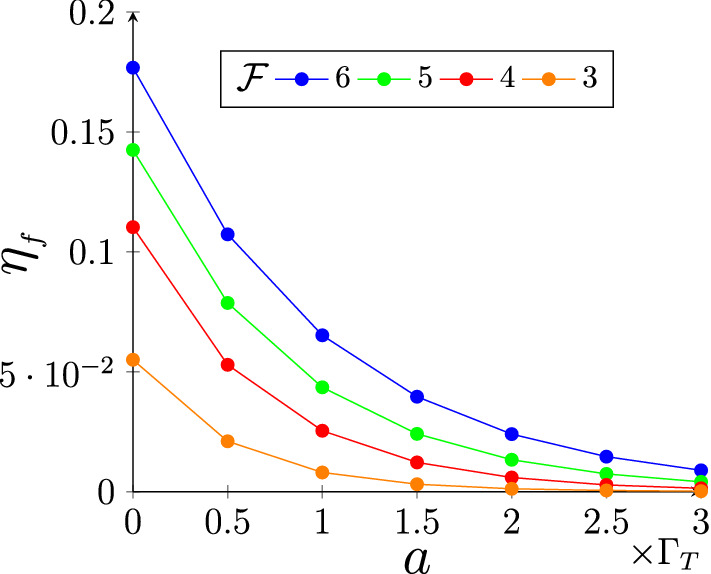
Here we have plotted the efficiencies as a function of the fluctuation strength *a* of the comb spacing and for various values of the finesse ${\mathcal {F}} = \Delta /(\gamma +2\Gamma _T)$. We have chosen the optical depth $\alpha L = 30$, tooth width $\gamma =5$MHz and the calculations are done at temperature $T = 10$K. From here we can see that although the efficiency is decreasing as we increase the randomness, the efficiencies are significant even at fluctuations of the order of Doppler broadening $\Gamma _T$.

**Figure 3:**
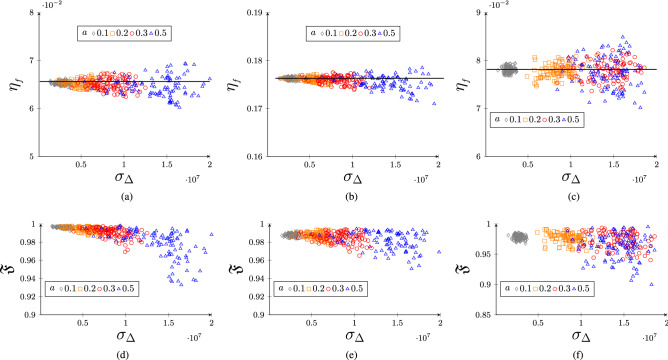
In (**a**, **b**) we plot the efficiency for non-uniform comb with no fluctuations in the tooth-spacing. Here the non-uniformity is quantified by the standard deviation $\sigma _\Delta$ of mean frequency of each of the tooth in the frequency comb. We have set ${\mathcal {F}}=3$, $\alpha L=18$ for (**a**) and ${\mathcal {F}}=6$, $\alpha L=30$ for (**b**), and the dashed line represents efficiency for the uniform comb. In (**c**) we plot efficiency of Cesium atom I-AFC at magnetic field strength $B=0.1T$. In (**d**–**f**) we plot the corresponding fidelities for the figures (**a**–**c**).

Although, the spacing between different hyperfine energy levels is predominately determined by the applied external magnetic field, the spin-orbit coupling and interactions with the nuclear spins can result in non-uniform spacing between these energy levels. This non-uniformity can make a frequency comb unusable for purpose of quantum memory. Furthermore, when we dope these atoms in some dielectrics, or due to stray electric and magnetic fields, there can be fluctuations in the mean frequencies of different transitions. In this section, we address the effect of these adversaries on the efficiency of the I-AFC based quantum memory.

We start with fluctuations in the comb spacing, which is introduced by shifting the comb spacing by a random number $\zeta$ with probability $P(\zeta ) \propto \exp [-\zeta ^2/(2\sigma _\zeta ^2)]$. Here we have assumed the probability distribution to be Gaussian with width $\sigma _\zeta$. In this scenario, the propagator ${\mathcal {D}}$ reads

15 $$\begin{aligned} \mathcal {D}( \omega ,\zeta ) = \sum _{n=-3}^{3} \dfrac{\gamma \alpha _n}{\gamma /2 +\Gamma _T+ \mathrm {i}(n\Delta + \zeta _n +\omega )}+\dfrac{\mathrm {i}\omega }{c}. \end{aligned}$$

Following the approach given in Eq. (11) the averaged propagator is written as

16 $$\begin{aligned} \mathcal {D}( \omega ) = \sum _{n=-3}^{3} \dfrac{\gamma \alpha _n}{\gamma /2 +\Gamma _T+ a+\mathrm {i}(n\Delta + \omega )}+\dfrac{\mathrm {i}\omega }{c}, \end{aligned}$$

where $2a\propto \sigma _\zeta$ is the FWHM of the probability distribution. Eq. (16) shows that a general randomness in the comb spacing increases the broadening similar to the Doppler broadening. In Fig. 2 we plot the forward efficiencies as a function of width *a* for different values of finesse ${\mathcal {F}}$. Here $\Gamma _T ~(\sim 5 \times 10^7) \gg \gamma ~(\sim 5 \times 10^6)$ thus making the Doppler broadening dominant over natural broadening and we take the randomness *a* as a multiple of the Doppler width $\Gamma _T$. We observe that the efficiency drops with increasing randomness. However, increasing the finesse results in higher efficiency; hence, the effect of the fluctuating comb spacing can be mitigated by applying stronger magnetic field. Interestingly, the effect of random fluctuations on the Fidelity between the input and output states is negligible. This is because the random fluctuations in the comb spacing effectively increases the tooth width which does not affect the phases in the outcome electric field.

Next, we consider the case when the frequency comb is not uniform but there is no fluctuations in the mean frequencies. For such systems the propagator $\mathcal {D}$ can be written as

17 $$\begin{aligned} \mathcal {D} = \sum _{n=-3}^{3} \dfrac{\gamma \alpha _n}{\gamma /2 +\Gamma _T+\mathrm {i}(\delta _n+ \omega )}+\dfrac{\mathrm {i}\omega }{c}. \end{aligned}$$

For simplicity, we can assume the frequency $\delta _n$ of each of the tooth as a deviation from a average position, i.e., $\delta _n = n\Delta + \zeta _n$, where $\zeta _n$ is fixed and sampled randomly from the set $[-a\Gamma _T,a\Gamma _T]$. We use the standard deviation in the mean frequency of each of the tooth of the frequency comb as the measure for the non-uniformity in the frequency comb. The standard deviation can be calculated using the following expression

18 $$\begin{aligned} \sigma ^2_\Delta =\frac{1}{N-1} \sum _{n=-3}^3 {\zeta ^2_n}-\langle \zeta \rangle ^2, \end{aligned}$$

where *N* is the number of teeth.

In Fig. 3a and b we plot the numerically optimized efficiencies as a function of standard deviation in comb spacing for different finesse. Since multiple non-uniform combs can have the same value of the standard deviation $\sigma _\Delta$, we can have a band of efficiencies for the same $\sigma _\Delta$. We see that this band broadens with the standard deviation $\sigma _\Delta$ which itself is a function of *a*. An interesting finding of this plot is that occasionally large $\sigma _\Delta$ can result in higher efficiencies than the perfectly uniform combs. In other words, the uniform combs are not optimum for efficient quantum memory. Although there is no obvious explanation for this increase in the efficiency, we noticed that for some of these cases the average finesse is also larger than the ideal comb. Larger finesses is known to improved efficiency. For example, if we choose fluctuations from the set $[0,a\Gamma _T]$ for the teeth that are on the right side of the center of the frequency comb and from the set $[-a\Gamma _T,0]$ for the teeth on the left of the center such that the comb is symmetric about the center, we always observe an improved efficiency. In Fig. 3c and d we plot the fidelity as a function of $\sigma _\Delta$. Unlike the efficiency, the variation in the fidelity is not too severe.

As an example of a real system we consider the I-AFC in Cesium atom. At temperature 10*K* and magnetic field $B=0.1T$ the maximum efficiency we can achieve in this system is $\eta _f\approx 7.8\%$. In Fig. 3c and f we plot the optimized efficiency and the fidelity in Cs I-AFC as a function of $\sigma _\Delta$ upon introducing the fluctuations in the comb. We have set ${\mathcal {F}}=3$ and $\alpha L=18$ and observe that with the increase in the randomness the band for the fidelity as well as efficiency broadens and in some cases the efficiency can reach upto $8.5\%$.

### Random and non-uniform optical depths

**Figure 4:**
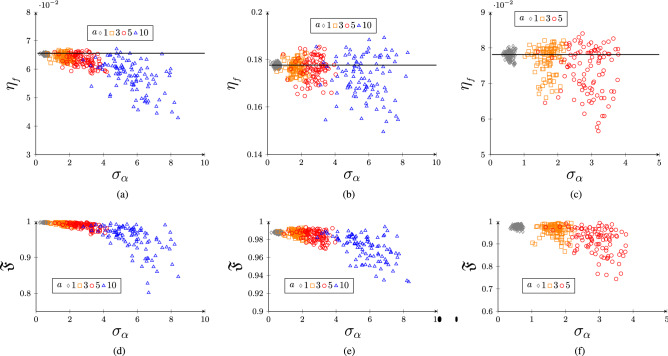
(Color online) In (**a**) (${\mathcal {F}}=3$, $\alpha L=18$), (**b**) (${\mathcal {F}}=6$, $\alpha L=30$) and (**c**) (Cesium at 0.1T) we plot efficiency and standard deviation of optical depths over 100 trails where dashed line represents efficiency with zero randomness. In (**d**–**f**) we polt the corresponding fidelities.

The optical depth of various teeth in I-AFC in natural atoms is non-uniform in general. The non-equal transition probability between different atomic states is one of the biggest contributor to such non-uniformity. This can be further exaggerated by random environmental effects. In this section we study the effect of non-uniform optical depth and fluctuation therein of different teeth of the I-AFC on the efficiency of the quantum memory. Similar to Section “Background”, we incorporate the fluctuating optical depth in I-AFC dynamics by adding randomness in the propagator ${\mathcal {D}}$ as follows

19 $$\begin{aligned} \mathcal {D} = \sum _{n} \dfrac{(\gamma \alpha _n+\zeta _n)}{\gamma /2 +\Gamma _T+ \mathrm {i}(n \Delta +\omega )}+\dfrac{\mathrm {i}\omega }{c}, \end{aligned}$$

where $\zeta _n$ determine the fluctuations in the optical depth and it occurs with probability $P(\zeta ) \propto \exp [-\zeta ^2/2\sigma _\zeta ^2]$. The average propagator can be calculated by taking average of Eq. (19) with the probability function $P(\zeta )$. Since the probability is an even function of $\zeta$, from Eq. (13) we can see that the randomness has no effect on the optical depths. This shows that the fluctuating optical depths do not affect the efficiency of the quantum memory.

However, the non-uniform optical depth without fluctuations can result in the lower quality of the memory. In this case, the propagator for such systems is defined by Eq. (19) but there is no probability distribution over $\zeta$. But $\zeta _n$ is sampled from the set $[-a,a]$. The non-uniformity of the comb is quantified by the standard deviation which is defined as $\sigma ^2_\alpha =1/(N-1) \sum _{n=-3}^3 {\zeta ^2_n}-\langle \zeta \rangle ^2$ for *N* number of teeth.

In Fig. 4 we plot the efficiency and the fidelity as a function of standard deviation in optical depth for different finesse. The calculations are done at temperature $T=10$K. The results for non-uniform optical depth are qualitatively similar to the one we got for non-uniform comb spacing. In this case also we see the non-uniform comb occasionally yield more efficient quantum memory as oppose to uniform comb. This might be because of the occasional average increase in the absorption strength of the frequency comb.

In Fig. 4c and f we plot the efficiency and fidelity in Cesium atom as a function of $\sigma _\alpha$. Here we have chosen $({\mathcal {F}}=3,\alpha L=18)$, temperature to be 10K, and external magnetic field 0.1T. As expected, the efficiency and the fidelity in Cs atoms are robust against a large fluctuation in $\alpha$.

## Conclusion

In conclusion, we have shown that the I-AFC based quantum memory is robust against non-uniformity in the comb spacing, and non-uniform and fluctuating optical depths. The fluctuations in the comb spacing can affect the quantum memory efficiency in a strong way. However, this effect can be easily mitigated by increasing the finesse of the I-AFC, which can be done by applying stronger magnetic field. Our study shows that even the imperfect atomic systems in the extreme environmental conditions can be used for efficient I-AFC based quantum memories highlighting the robust nature of I-AFC based quantum memory.
